# Zebrafish *Endzone* Regulates Neural Crest-Derived Chromatophore Differentiation and Morphology

**DOI:** 10.1371/journal.pone.0002845

**Published:** 2008-07-30

**Authors:** Brigitte L. Arduini, Glen R. Gallagher, Paul D. Henion

**Affiliations:** 1 Center for Molecular Neurobiology, Ohio State University, Columbus, Ohio, United States of America; 2 Department of Neuroscience, Ohio State University, Columbus, Ohio, United States of America; Temasek Life Sciences Laboratory, Singapore

## Abstract

The development of neural crest-derived pigment cells has been studied extensively as a model for cellular differentiation, disease and environmental adaptation. Neural crest-derived chromatophores in the zebrafish (Danio rerio) consist of three types: melanophores, xanthophores and iridiphores. We have identified the zebrafish mutant *endzone* (*enz*), that was isolated in a screen for mutants with neural crest development phenotypes, based on an abnormal melanophore pattern. We have found that although wild-type numbers of chromatophore precursors are generated in the first day of development and migrate normally in *enz* mutants, the numbers of all three chromatophore cell types that ultimately develop are reduced. Further, differentiated melanophores and xanthophores subsequently lose dendricity, and iridiphores are reduced in size. We demonstrate that *enz* function is required cell autonomously by melanophores and that the *enz* locus is located on chromosome 7. In addition, zebrafish *enz* appears to selectively regulate chromatophore development within the neural crest lineage since all other major derivatives develop normally. Our results suggest that *enz* is required relatively late in the development of all three embryonic chromatophore types and is normally necessary for terminal differentiation and the maintenance of cell size and morphology. Thus, although developmental regulation of different chromatophore sublineages in zebrafish is in part genetically distinct, *enz* provides an example of a common regulator of neural crest-derived chromatophore differentiation and morphology.

## Introduction

The neural crest is a transient vertebrate embryonic cell population that gives rise to a wide variety of cell types, including chromatophores, craniofacial cartilage, and neurons and glia of the peripheral nervous system [1]. This array of neural crest-derived cell types has long been of interest in studying the mechanisms of cell diversification among embryonic cell populations. The development of neural crest-derived chromatophores in particular has been studied extensively, and many important mechanistic insights have resulted from the analysis of mouse and zebrafish mutants [1]–[5].

Vertebrate chromatophore populations are readily observed, as they produce their own visible intrinsic markers. In addition, chromatophores are not strictly required for viability [5]–[7]. As a result, chromatophores have long been used to study developmental processes such as cell fate specification, proliferation, migration, differentiation, and survival. Mice and other mammals have a single chromatophore cell type termed melanocytes [8]. Hundreds of mouse coat color mutants have been identified, covering over 100 loci, which affect multiple cellular processes [4], [5]. Further, many of these mutations in mice have proved to be medically relevant as models for human diseases involving the same genes [9]. Besides the melanocytes (melanophores) also found in mammals, zebrafish and other ectotherms possess neural crest-derived yellow xanthophores and iridescent iridiphores [10], [11]. In addition to the isolation of several zebrafish chromatophore mutants that arose spontaneously [12], [13], numerous mutagenesis screens have yielded over 100 mutations affecting various processes in the development of different combinations of the chromatophore types [6], [14]–[17].

Studies from several vertebrates, including zebrafish, have led to the extensive characterization of melanophore development, and to a lesser extent, xanthophore and iridiphore development [2], [4], [8], [18], [19]. Prior to overt differentiation, chromatophore precursors are referred to as chromatoblasts, and can be identified by expression of genes specific to one or multiple chromatophore sublineages. Sox10, mutations in which cause Waardenburg-Hirschsprung Syndrome in humans, is required for development of nonectomesenchymal neural crest derivatives, including all chromatophores, as well as many peripheral neurons and glia [20], [21]. Sox10 has been shown to directly regulate expression of *microphthalmia-associated transcription factor* (*mitf*), which is both necessary and sufficient for melanophore development, and *dopachrome tautomerase* (*dct*), an enzyme in the melanin synthesis pathway [6], [22]–[24]. The receptor tyrosine kinase *c-kit* is also expressed by melanoblasts, and appears to be necessary for their migration and survival [25], [26]. Similarly, the *kit* ortholog *fms*, which has no known role in mammalian melanocyte development, is required for the migration of embryonic xanthophores and the specification of a subset of adult melanophores in zebrafish [27], [28]. *fms* is expressed by embryonic xanthoblasts and macrophages, which can be distinguished from one another based on location and cellular morphology [28]–[30]. Synthesis of yellow pteridine pigments, found in xanthophores, requires *xanthine dehydrogenase* (*xdh*), which is correspondingly expressed by xanthoblasts [28], [31], [32]. Both *fms* and *xdh* are co-expressed in a subset *mitf^+^* cells in the premigratory neural crest, which may represent uncommitted precursors of melanophores or xanthophores [28]. Neither of these genes is co-expressed with *c-kit*, however, and both have been used as specific diagnostic markers of xanthoblasts at migratory and post-migratory stages of chromatophore development [28], [33]. The enzyme GTP-cyclohydrolase (Gch) is involved in the conversion of intermediates of both melanin and pteridine synthesis [34]–[36]. Accordingly, *gch* expression is observed in both melanoblasts and xanthoblasts [28]. A G protein-coupled receptor, *endothelin receptor* (*ednr*) *b*, is also expressed by neural crest-derived chromatophore precursors [37]–[41] Homozygous *ednrb* mutant mice are almost completely devoid of melanocytes [4], [5]. In contrast, zebrafish *ednrb1/rose* mutants display defects in subsets of adult melanophores and iridiphores but lack an embryonic chromatophore phenotype [37]. In the zebrafish embryo, *ednrb1* is initially expressed by all chromatophore sublineages, but by late embryonic/early larval stages, is restricted to iridiblasts and iridiphores [37].

Morphologically, differentiated melanophores and xanthophores are large and dendritic with many processes, while iridiphores are rounded in shape [8]. In ectotherms, considerable attention has been given to mechanisms of color adaptation, reversible changes in pigmentation brought on by prolonged exposure to either light or dark environments [42]. Extensive analyses, especially in a variety of fish species, have revealed that this occurs through relocalization of pigment organelles within cells, changes in cell morphology, and proliferation and apoptosis of chromatophores [42]–[45]. In adults, these processes appear to be under hormonal, as well as nervous control [43]–[45]. α-melanophore-stimulating hormone (α-MSH) and melanin-concentrating hormone (MCH) appear to have mutually antagonistic effects on melanophores, with α-MSH enhancing melanin and melanophore development, and MCH promoting aggregation of melanosomes and downregulating secretion of α-MSH [46]–[49]. The effects of α-MSH are enacted in part through influence on *mitf* expression in mammals [50]–[52]. Rho GTPase-mediated cytoskeletal rearrangements may play roles in redistribution of pigment organelles and dendrite collapse [42].

Despite these and other data that have been amassed regarding chromatophore development in a variety of vertebrate systems, many questions remain. For example, relatively few genes have been identified that are required for the development of all embryonic chromatophores, and yet are specific to chromatophore sublineages within the neural crest. For example, Sox10 is necessary for development of chromatophore sublineages, but is also required for some crest-derived neurons and glia, and mammalian *ednrb* is necessary for both melanocyte and enteric neuron development [39], [53]–[57]. Further, although expressed by all three zebrafish chromatophore cell types, *ednrb1* appears to be dispensable for their embryonic development [37]. While hormonal and neuronal influences have been demonstrated for pigment distribution, cell morphology, proliferation and survival of differentiated chromatophores, less is known about the downstream effectors governing these processes. Additionally, these studies focus on adults, and comparatively little is known about control of pigment cell morphology or color adaptation at embryonic stages.

We report here characterization of the zebrafish mutant *endzone^b431^* (*enz^b431^*;*enz*), which was isolated based on the abnormal appearance of embryonic chromatophores. Our results indicate that all three chromatophore types are similarly reduced in cell size and number in *enz* mutant embryos. We show that *enz* is required specifically for chromatophore sublineages within the neural crest, and that this requirement is cell autonomous. Further, we report the identification of multiple *enz* alleles and progress toward the molecular identification of the *enz* locus. We suggest that *enz* is a relatively late cue required by pigment cell sublineages of the neural crest during embryogenesis, and is indicative of common requirements of chromatophores that are distinct from other neural crest derivatives.

## Results

### Live phenotype of *endzone* mutants

**Figure 1:**
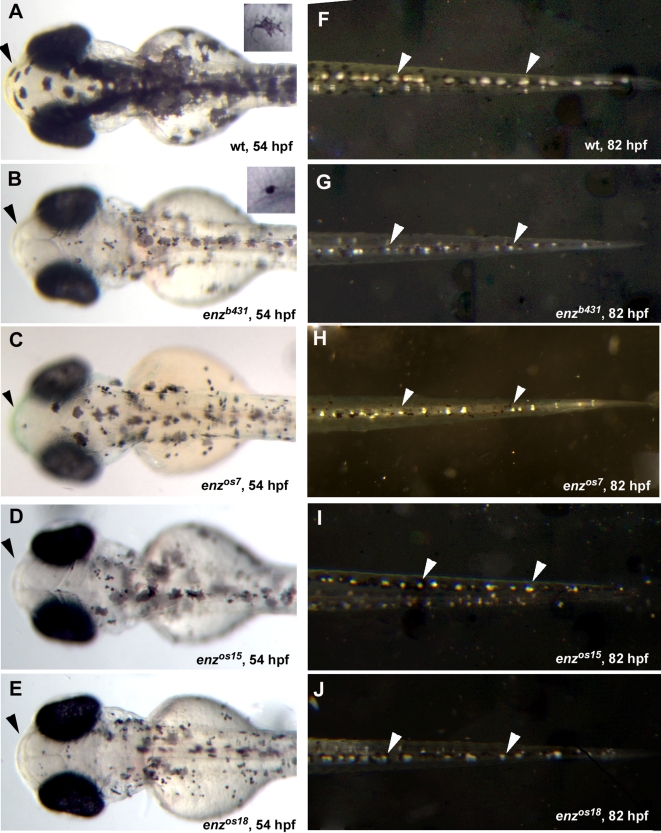
All three neural crest-derived chromatophore cell types are affected by *enz* mutations. Dorsal views of 54 hpf (A–E) and 82 hpf (F–J) wild-type (A, F) and *enz* mutant (B–E, G–J) embryos. (A, A inset) Wild-type melanophores are stellate and darkly pigmented at 54 hpf. (B–E) In contrast, *enz^b431^* (B), *enz^os7^* (C), *enz^os15^* (D) and *enz^os18^* (E) mutant embryos have small, punctate melanophores at this stage (compare insets in A and B). Yellow xanthophores, observed dorsally in the head of wild-type embryos (arrowhead in A) appear to be absent in *enz* homozygotes (B–E, arrowheads). (F–J) Iridescent iridiphores are also reduced in size in *enz* mutants (G–J, arrowheads) compared to wild-type siblings (F, arrowheads) at 82 hpf.

**Figure 2:**
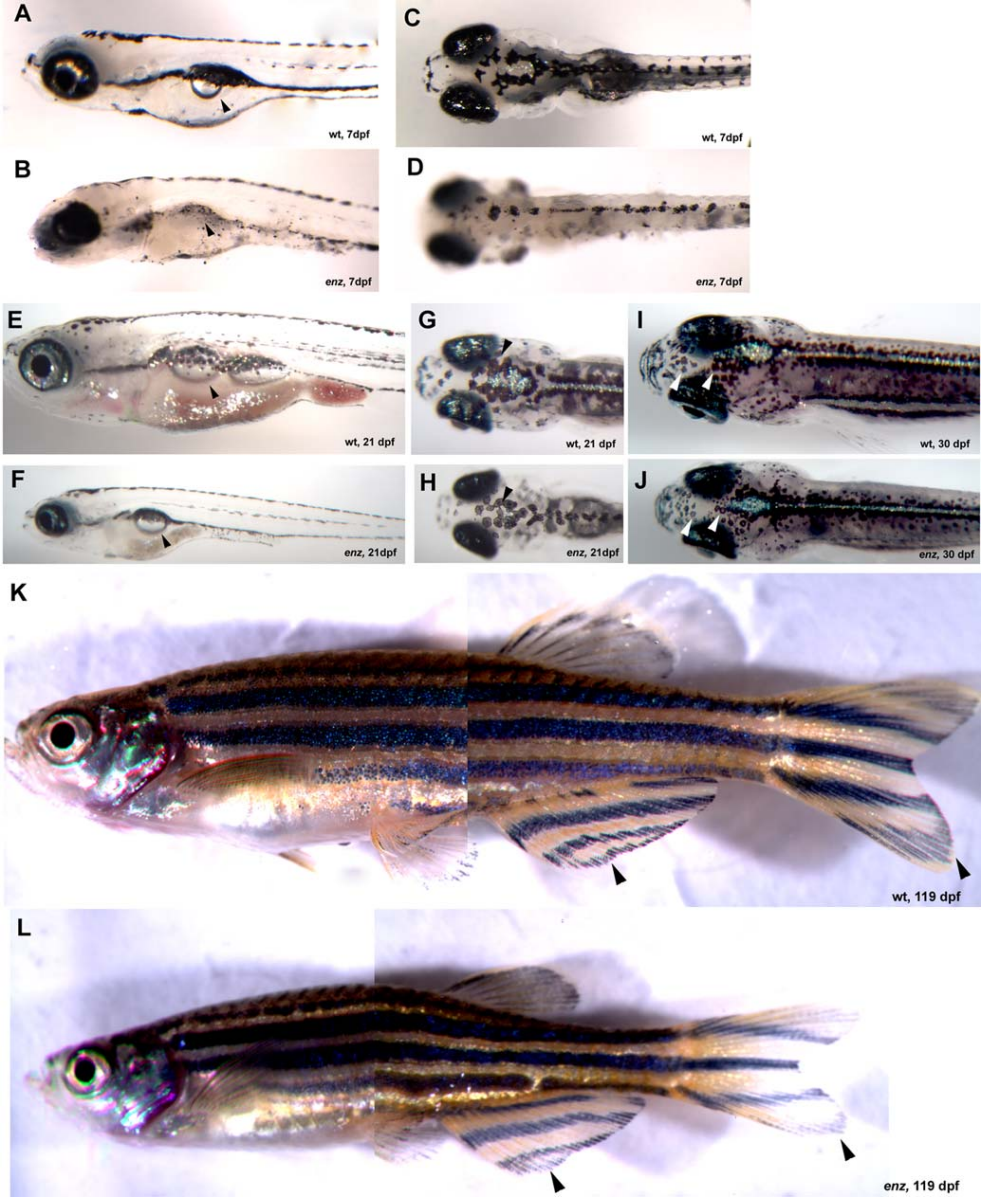
*enz* larvae and adults are undersized compared to wild-type siblings. Lateral (A, B) and dorsal (C, D) views of 7 dpf wild-type (A, C) and *enz* (B, D) larvae. (A) Wild-type zebrafish develop swim bladders between 4 and 6 dpf (arrowhead). (B) The vast majority of *enz* homozygous larvae do not develop swim bladders (arrowhead). Some melanophores recover in size and morphology in *enz* homozygotes (D) compared to wild-type siblings (C). (E–L) Those *enz* homozygous larvae that do develop swim bladders survive, but are runted compared to wild-type siblings (stage-matched wild-type and *enz* mutant larvae shown at the same magnification). Lateral (E, F) and dorsal (G–J) views of wild-type (E, G, I) and *enz* mutant (F, H, J) larvae at 21 dpf (E–H) and 30 dpf (I, J). Melanophores continue to be paler in *enz* mutant larva than in wild-type siblings through at least 30 dpf (see arrowheads in G–J). (K, L) Lateral views of 119 dpf wild-type (K) and *enz* homozygous (L) adults. Normal overall morphology and pigmentation of *enz* mutant adults, as well as nascent fin stripe formation (L, arrowheads), suggests generalized growth retardation in *enz* mutants compared to wild-type siblings.

*enz* was identified in a chemical (ethyl-nitrosourea, ENU) mutagenesis screen for mutations affecting neural crest derivatives as previously described [15]. The *enz* mutant was identified based on its altered pigment pattern. At 54 hours post-fertilization (hpf), the morphology of melanophores is dramatically altered being smaller and less stellate in *enz^b431^* mutant embryos compared to wild-type siblings (compare insets, Fig. 1A, B). In addition, the pigmented retinal epithelium (PRE) of *enz* homozygotes, which is not neural crest-derived, is pale compared to that of wild-type siblings prior to 48 hpf. Unlike crest-derived melanophores, however, the PRE phenotype recovers rapidly (Fig. 1 and data not shown). At 54 hpf, *enz^b431^* embryos also lack the characteristic yellow cast caused by xanthophores concentrated dorsally in wild-type embryos (Figure 1). In addition, iridiphores are reduced in number and size in 82 hpf and 6 days-postfertilization (dpf) *enz* mutant larvae compared to wild-type siblings (Figure 1F-H, Table 1 and data not shown). Recently, we identified three additional ENU-induced *enz* alleles as determined by non-complementation of *enz^b431^* and linkage analysis (Figure 1 and data not shown). All four *enz* alleles are recessive. These alleles vary in the severity of the developmental defects in all three chromatophore phenotypes. *enz^os15^* is less severe than *enz^b431^*, *enz^os7^*, and *enz^os18^*, which are similar in expressivity. All data refer to *enz^b431^* unless otherwise specified. Most *enz* homozygotes do not develop swim bladders, and subsequently fail to survive past early larval stages (Figure 2A, B). *enz* mutant larvae that do develop swim bladders survive, but are runted compared to wild-type siblings through adulthood. Qualitative differences in melanophore pigmentation persist through at least 30 dpf (Figure 2). As adults, *enz* homozygotes are viable as both males and females, and are fertile (not shown), although they remain smaller than wild-type siblings.

**Table 1:** Melanophore and iridiphore numbers are reduced in enz mutant embryos.

Melanophores (4 dpf)		Iridiphores (6 dpf)	
Wt	23.9±0.7	wt	53.1±0.9
*Enz*	17.7±1.1	*enz*	41.4±1.7
compared to wt	74.1%	compared to wt	78.0%
	P<0.0001		P<0.0001
Larvae were mounted dorsally. Melanophores were counted in the dorsal and ventral stripes from somites 5–14. The mean number of cells in wild-type and *enz* embryos were subjected to a one-tailed T-test to determine whether observed differences were significant.		Larvae were mounted dorsally. Iridiphores were counted posterior of somite 1 in the dorsal stripe and posterior of the yolk sac in the ventral stripe. The mean number of cells in wild-type and enz embryos were subjected to a one-tailed T-test to determine whether observed differences were significant.	

### Within the neural crest lineage, *enz* selectively affects chromatophore development

**Figure 3:**
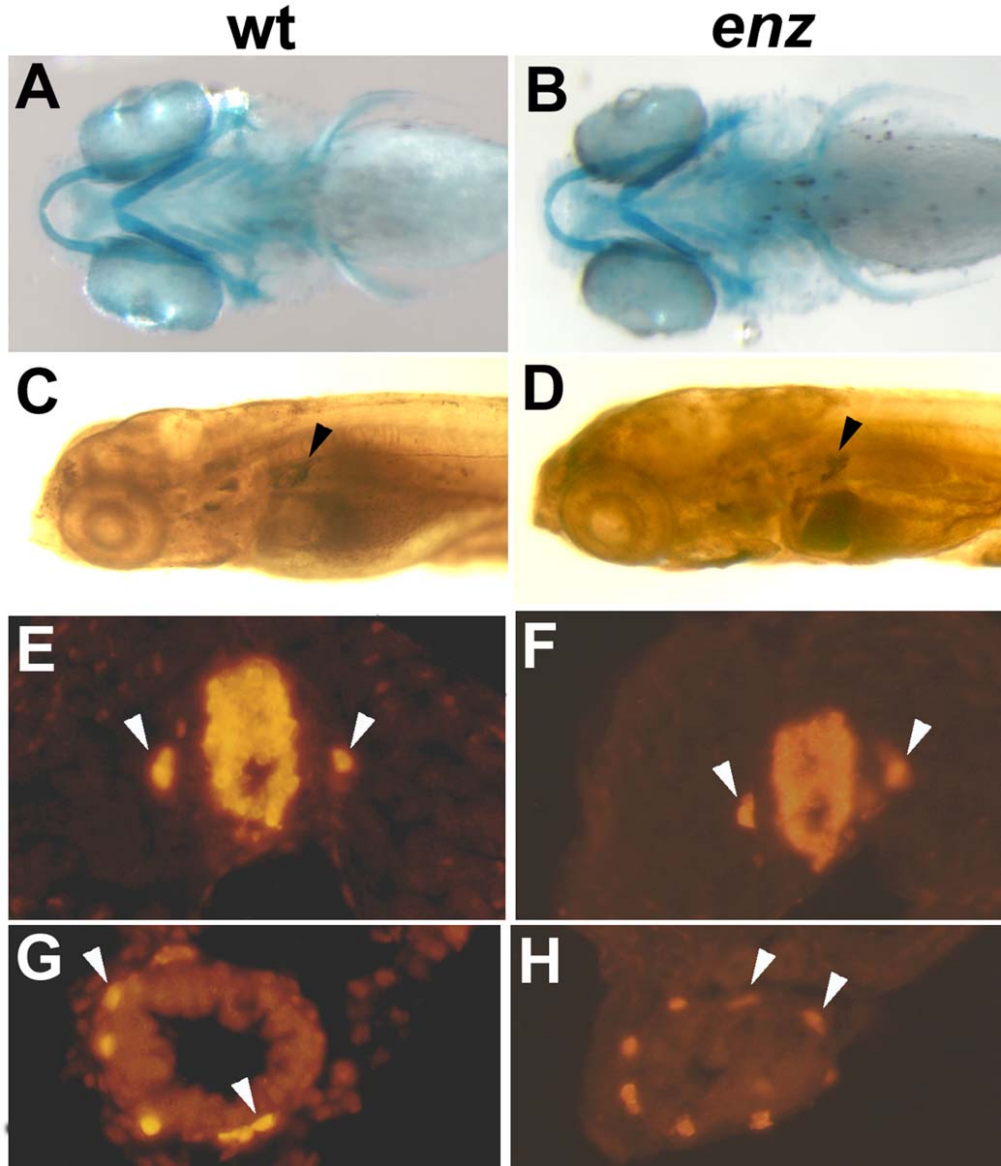
*enz* selectively affects chromatophores among neural crest derivatives. Wild-type (A, C, E, G) and *enz* (B, D, F, H) mutant embryos. Craniofacial cartilages revealed with alcian blue staining are normal at 5 dpf (A, B: ventral view). Cervical sympathetic neurons, which express TH immunoreactivity (arrowheads in C, D), are indistinguishable between wild-type (C) and *enz* mutant (D) embryos at 7 dpf. (E–H) Hu-positive neurons of the dorsal root ganglia (E, F, arrowheads) and the enteric nervous system (arrowheads in G, H) also appear normal in *enz* mutant embryos (F, H) compared to wild-type siblings (E, G) at 7 dpf.

**Figure 4:**
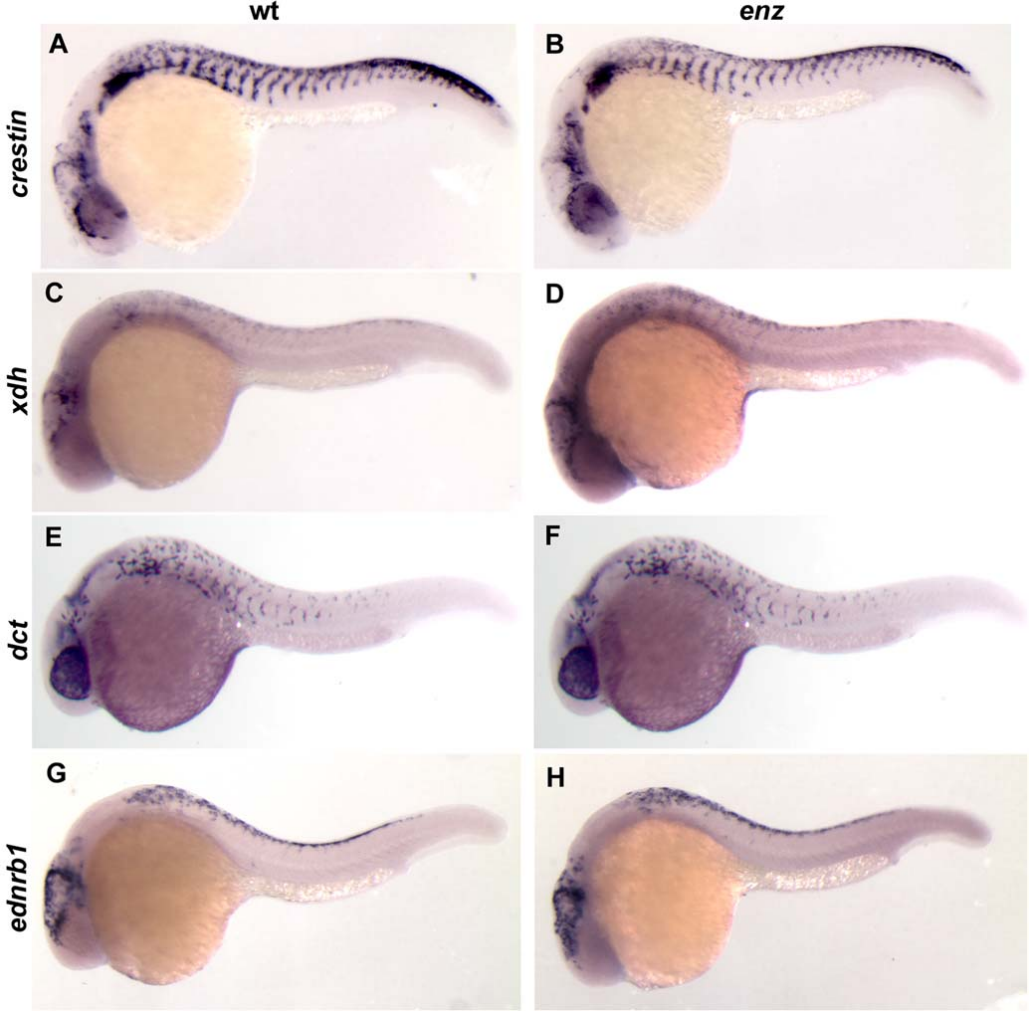
The numbers and distribution of chromatophore precursors appear normal in *enz* homozygotes at 24 hpf. Lateral views of 24 hpf wild-type (A, C, E, G) and *enz* mutant (B, D, F, H) embryos. Early neural crest cells (*crestin*; A, B), xanthoblasts (*xdh*; C, D), melanoblasts (*dct*; E, F) and all chromatophore precursors (*ednrb1*; G, H) are all qualitatively normal at this stage.

Because of the visible defects in neural crest-derived chromatophore development in *enz* mutant embryos, we investigated whether the development of other cell types derived from the neural crest were affected by the *enz* mutation. Molecular markers indicated that neural crest-derived peripheral neuron and cranial glial populations develop normally in *enz* homozygotes (Table 2). For example, cervical sympathetic neurons, enteric neurons, and neurons of the dorsal root ganglia are all present in qualitatively normal numbers and positions in *enz* mutant embryos (Figure 3 and data not shown). Craniofacial cartilage, stained with alcian blue, was also found to be normal in terms of individual elements and their shapes (Figure 3A, B). In addition, the pan-neural crest marker *crestin* was used to analyze neural crest populations at different embryonic stages. In wild-type embryos, *crestin* is first expressed in neural crest cells at the boundary of neural and non-neural ectoderm during gastrulation. Expression continues in premigratory and migratory neural crest cells, and persists until slightly after overt differentiation of neural crest derivatives, such that by 24 hpf, *crestin*-expressing cells are found throughout the embryo [58]. *crestin* expression is normal in *enz* embryos at all stages, indicating that neural crest induction and migration are unaffected by the *enz* mutation and that neither the proliferation or survival of early neural crest cells are overtly compromised by the *enz* mutation (Figure 4A, B and data not shown). Further, we examined the expression of genes that, within the neural crest, are expressed by precursors for melanophores, [23], xanthophores [28] and all three chromatophore cell types [37] at 24 hpf. Expression of each gene is qualitatively normal at this stage, suggesting that effects of the *enz* mutation on crest-derived chromatophores occur relatively late in the development of these cell populations (Figure 4C–H). These data indicate that within the embryonic neural crest lineage, *enz* is required specifically for chromatophore development and only after neural crest dispersal and initial differentiation have largely occurred.

**Table 2:** Other neural crest derivatives are normal in *enz* mutant embryos.

Neural crest-related population	Marker	Stage	Phenotype?
Early neural crest cells	*crestin*	14s-24hpf	no
Enteric neurons	anti-Hu/location	7 dpf	no
Dorsal Root Ganglion Neurons	anti-Hu/location	7 dpf	no
Sympathetic neurons	anti-TH/location	7 dpf	no
Neural crest-derived glia	*foxd3*	48 hpf	no
Craniofacial cartilage	alcian blue	5 dpf	no

### Reduced numbers of differentiated chromatophores in *enz* mutant embryos

**Figure 5:**
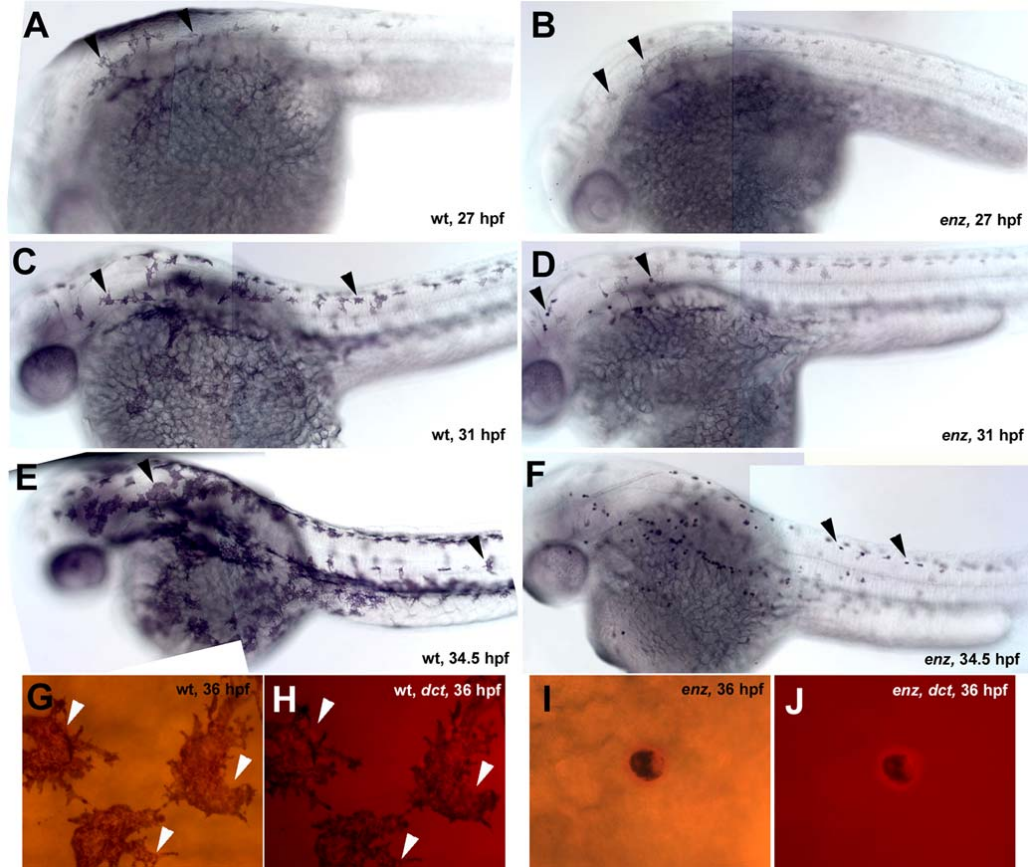
Melanophore cell morphology changes in *enz* mutant embryos. (A, B) At 27 hpf, wild-type melanophores are large, stellate and well-pigmented (A). *enz* melanophores are also large and stellate at this stage, but are pale compared to wildtype (B). (C, D) By 31 hpf, *enz* melanophore begin to transition to a punctate morphology (D), while wild-type melanophores remain large with many processes (C). (E, F) Wild-type (E) and *enz* mutant (F) embryos at 34.5 hpf. The morphological transition of melanophores in *enz* homozygotes continues in a rostro-caudal wave and is complete by approximately 48 hpf. (G–J) At 36 hpf, melanosomes are distributed throughout the cytoplasm of wild-type melanophores, reflecting the stellate morphology of these cells (G). (H) *dct* mRNA (red) is likewise distributed in the extensive processes of wild-type cells (arrowheads). Punctate distribution of melanosomes (I) and *dct* mRNA (J, red) in *enz^os18^* mutant melanophores is identical at 36 hpf reflecting cell shape change.

To determine whether appropriate numbers of chromatophores are generated in *enz* mutant embryos, we counted differentiated melanophores and iridiphores in *enz* homozygotes and wild-type siblings. At 4 dpf, the number of melanophores in *enz* mutant embryos is significantly reduced compared to wild-type siblings (P<0.0001, Table 1). Likewise, iridiphore numbers are reduced in *enz* homozygotes compared to wild-type siblings at 6 dpf (P<0.0001, Table 1). In both cases, only about 75% of the wild-type complement of each class of chromatophores is present in *enz* mutants at the stages examined. Quantification of xanthophore numbers was precluded by indistinct boundaries and overlap between these cells. However, methylene blue staining (see below) at 3 dpf, as well as *fms* expression between 48 and 53 hpf, revealed that while many xanthophores are present in *enz* homozygotes, their numbers are reduced in a manner qualitatively similar to that of melanophores and iridiphores (Figure 5 and data not shown). Thus, *enz* mutations appear to result in similar reductions in the numbers of differentiated chromatophores, while other neural crest-derived cell types are unaffected.

### Chromatophore cell morphology is altered in *enz* mutant embryos

**Figure 6:**
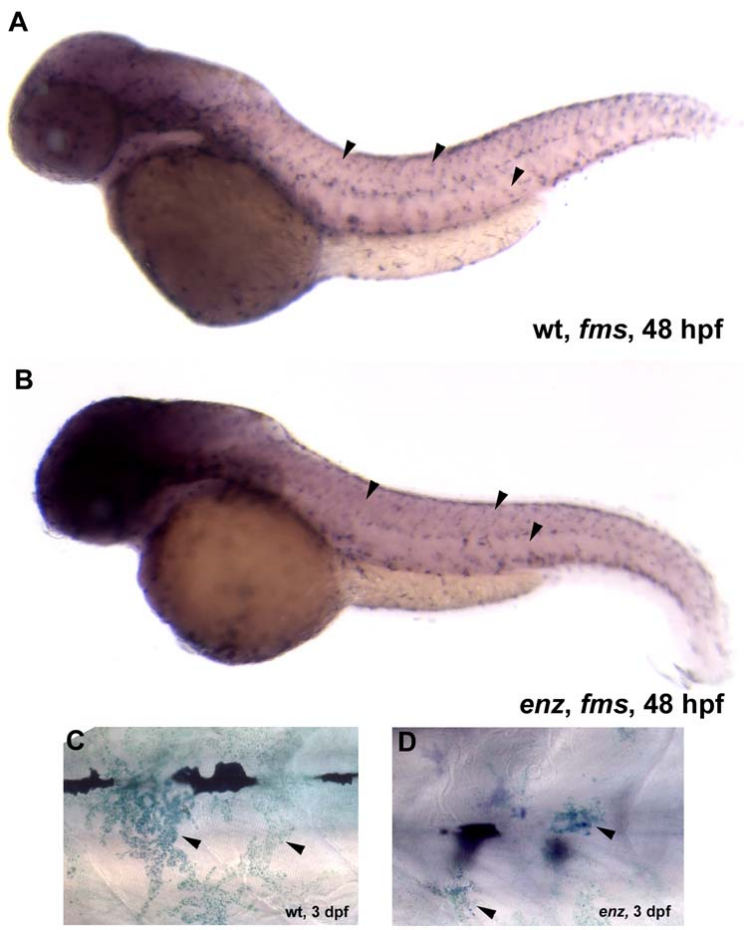
Xanthophores are qualitatively reduced in number and size in *enz* mutants. (A, B) *fms* expression in 48 hpf wild-type (A) and *enz* mutant (B) embryos. Qualitatively reduced *fms* expression suggests that fewer xanthophores are present in *enz* mutants than in wild-type siblings at this stage (arrowheads in A and B). (C, D) Methylene blue-stained xanthophores appear much larger in wild-type (C) embryos than in *enz* mutants (D) at 3 dpf.

In wild-type zebrafish embryos raised at 28.5°C, neural crest-derived melanophores normally begin to differentiate at approximately 25 hpf, while xanthophores and iridiphores begin to overtly differentiate at about 42 hpf and 72 hpf, respectively [59]. By 27 hpf, primarily in anterior regions, large, stellate, dark melanophores are present in wild-type embryos. In *enz* mutant embryos, as in wild-type siblings, melanophores differentiate at ∼25 hpf (data not shown). At 27 hpf, *enz* melanophores are stellate, but pale compared to those of wild-type siblings (Figure 5A, B). After 27 hpf, *enz* melanophores begin to transition from the initial pale, stellate appearance to a dark, punctate form, while wild-type melanophores remain stellate and dark. The transformation of melanophores occurs in a rostro-caudal wave, and is complete by about 48 hpf (Figure 5C–F and data not shown). We hypothesized that this apparent change in cell morphology of *enz* melanophores might be the result of either redistribution of melanosomes within cells or of a change in cell shape. To distinguish between these two possibilities, we performed *in situ* hybridization of melanized 36 hpf and 48 hpf wild-type and *enz* mutant embryos, using the melanophore sublineage-specific riboprobes *c-kit* [7] and *dct* [23]. In wild-type embryos, *c-kit* and *dct* mRNAs are distributed throughout the cell cytoplasm, including in the processes, reflecting stellate cellular morphology (Figure 5G, H and data not shown). The distribution of *dct* and *c-kit* mRNAs is punctate in *enz* melanophores, similar to the distribution of melanin (Figure 5I, J and data not shown). This is consistent with a cell morphology change, rather than only relocalization of melanin or melanosomes within a stellate cell. Subsequently, we quantified the area of punctate melanophores in *enz* mutant embryos compared to stellate melanophores in wild-type siblings. At 2 dpf, wild-type melanophores at cephalic levels have a mean area of 282.1±19.1 µm^2^, while *enz* melanophores at similar axial levels have a mean area of 21.9±1.7 µm^2^ (P<0.0001, Table 3). The change in melanophore cell morphology in *enz* mutant embryos further suggested that the apparent absence of yellow pigmentation might be due at least in part to a reduction in size of xanthophores in these embryos. Individual xanthophores are difficult to distinguish, and even *fms* and *xdh* expression appear as diffuse staining over the dorsal aspect of the embryo, precluding the quantitative type of analysis performed on melanophores (Figure 6A, B) [11], [14], [28]. However, qualitative observations of xanthophore morphology were made using methylene blue, which is taken up specifically by xanthophores and is concentrated around active pterinosomes, the organelles that produce pteridine pigments [60]. Methylene blue staining revealed that while some xanthophores are present in *enz* homozygotes, these appear much smaller and less stellate than xanthophores in wild-type siblings at 3 dpf (Figure 6C, D). Similarly, iridiphores are reduced in size in *enz* mutant embryos compared to wild-type siblings. In contrast to melanophores and xanthophores, iridiphores in the trunk of wild-type embryos have a rounded, rather than stellate morphology at 72 hpf (see Figure 1). While this is also true in *enz* homozygotes, overall iridiphore cell size, as measured by area, is reduced compared to wild-type siblings. At 6 dpf, the average iridiphore area in *enz* mutant larvae is reduced by ∼40% compared to that in wild-type siblings (P<0.0001, data not shown). Together, these data indicate that mutations in *enz* similarly affect all three neural crest-derived chromatophore types with respect to cell size and also result in loss of the the typical stellate morphology of melanophores and xanthophores.

**Table 3:** Melanophore size is reduced in *enz* mutant embryos at 2 dpf.

Melanophore Areas (µm^2^)	
wt	282.1±19.1
*enz*	21.9±1.7
compared to wt	7.8%
	P<0.0001
Larvae were mounted dorsally, observed on a Zeiss compound microscope, and photographed using Zeiss software. Zeiss software was then used to calculate the approximate areas of individual melanophores. The mean areas of cells in wild-type and *enz* mutant embryos were subjected to a one-tailed T-test to determine whether observed differences were significant.	

### *enz* acts cell autonomously with respect to melanophore development

**Figure 7:**
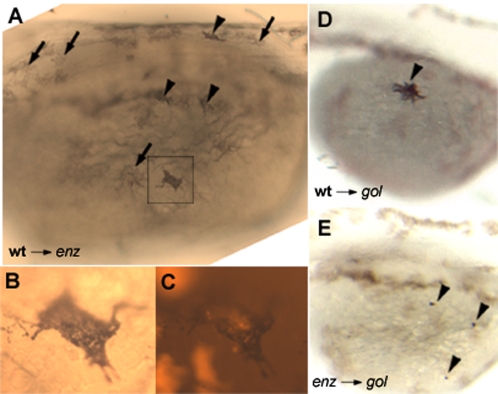
*enz* acts cell autonomously with respect to melanophore development. (A) Nomarski image of a 28 hpf *enz* mutant host that has received cells from a wild-type donor (anterior to the left). Several large, dark melanophores (arrowheads) are present in addition to pale melanophores (arrows) normally observed in *enz* mutant embryos at this stage (see also Figure 5B). (B) High magnification of a wild-type cell (boxed area in (A) that formed a dark melanophore in the mutant environment. (C) Melanophore in (B) viewed under a rhodamine filter. (D) Wild-type cells form dark, stellate melanophores in 48 hpf *gol* hosts. (E) *enz* cells give rise to punctate melanophores in 48 hpf *gol* hosts.

Mosaic analysis can be used to determine whether a mutation acts cell autonomously or non-autonomously, and thus predict the mode of action of a gene product within developmental pathways. Genetic mosaics were created between wild-type, *enz* mutant, and *golden^b1^* embryos. To facilitate the identification of donor-derived melanophores, particularly from *enz* donors, we utilized hosts homozygous for the *golden^b1^* mutation, which results in pale melanophores that are distinct from wild-type melanophores [7], [61]. Donor embryos were labeled with lysinated rhodamine dextran (LRD). Cells from donor embryos were then transplanted into unlabeled hosts, in which chromatophore development was subsequently observed. LRD-labeled cells from wild-type embryos formed dark, stellate melanophores at 28 hpf when transplanted into *enz* hosts (n = 6; Figure 7). In the reciprocal experiment, LRD-labeled cells from wild-type or *enz* mutant embryos were transplanted into unlabeled *golden^b1^* homozygous hosts [7], [61]. While wild-type cells were able to form dark, stellate melanophores (n = 13; Figure 7D), *enz* mutant cells formed punctate melanophores (n = 5; Figure 7E). Together, these data demonstrate that *enz* acts cell autonomously with respect to melanophore development, and inferentially, may act in a similar fashion in the development all chromatophores.

### The *enz* locus is located on chromosome 7

As an initial step towards cloning the *endzone* gene, we placed allele *enz^b431^* on the zebrafish linkage map [62], [63]. Simple sequence length polymorphisms [64], [65] between AB* and WIK zebrafish strains were used to map *enz* to chromosome 7. Parthenogenetic diploid embryos were generated by suppressing the second meiotic division using early pressure [66]. *enz* and wild-type embryos were identified by live phenotype, and PCR was performed on DNA from individual *enz* and wild-type embryos. The *enz* locus was initially localized to chromosome 7 in EP diploid embryos using three markers, z10441, z11652 and z6819 (see Methods). Subsequent recombination analysis employing DNA from 889 individual haploid embryos established a tentative interval in which *enz* lies proximal to z8252 and z10441 and distal to z55887 and z6819.

### Analysis of compound *trpm7/enz* mutants supports a late role for *enz* in chromatophore development

To investigate the role of *enz* compared to a known gene, we generated a line of fish doubly heterozygous for *enz^b431^* and *trpm7^b508^*, a gene previously shown to be required for development of the majority of neural crest-derived melanophores [67]–[69]. *trpm7^b508^* appears to affect melanophore development prior to overt differentiation, as most melanophores fail to appear in *trpm7^b508^* homozygotes. Further, there is evidence that some melanophore precursors undergo apoptosis in *trpm7^b508^* mutant embryos [67]. Those melanophores that do develop in *trpm7* single mutant embryos have punctate cell morphology upon differentiation, in contrast to the transiently stellate cell morphology of *enz* melanophores. In clutches obtained from double heterozygote crosses, four classes of phenotypes were observed. As expected, three of these classes were wild-type, *trpm7* single mutants and *enz* single mutants. The fourth phenotypic class, approximately 1/16 of the total number of embryos, exhibited defects resembling both *trpm7* and *enz* mutant embryos, and is presumed to represent double mutants. *trpm7-enz* double mutants exhibit an early reduction in melanophore number as well initial punctate melanophore morphology that is characteristic of the phenotype of *trpm7* single mutants. Iridiphores and xanthophores, on the other hand, are depleted as in *enz* homozygotes. At approximately 65 hpf, double mutants are touch insensitive, as are *trpm7* mutants. Thus, double mutants show a combination of the *trpm7* and *enz* phenotypes. Depletion of melanophores and initially abnormal melanophore morphology early in double mutants as in *trpm7* homozygotes supports the inference that *trpm7* is epistatic to *endzone* during melanophore development.

## Discussion

### Zebrafish *enz* is selectively required during neural crest development for the terminal differentiation and maintenance of cell morpholgy in late embryonic and larval chromatophores

The zebrafish mutant *enz* was identified based on the reduced size and numbers of neural crest-derived melanophores, xanthophores and iridiphores. Analysis of the development of other neural crest derivatives indicates that the *enz* mutation selectively affects chromatophore sublineages, as non-chromatophore derivatives, based on diagnostic gene expression, are morphologically indistinguishable from wildtype embryos. Whether all non-chromatophore derivatives are functionally normal in *enz* mutants, however, is not known. We observed that melanophores begin to differentiate in *enz* mutant embryos at approximately 25 hpf, similar to wild-type siblings. However, these melanophores were pale compared to wild-type melanophores, and later transitioned to a punctate morphology due to a change in cell shape as opposed to only a change in melanosome distribution. Wild-type melanophores, on the other hand, remain highly dendritic. Iridiphores and xanthophores in *enz* mutants also begin to differentiate normally in *enz* mutants but subsequently, like melanophores, appear smaller or less dendritic, respectively, and less chromatic than the same cell types in wild-type embryos. In addition, expression of genes diagnostic of early neural crest cells, melanoblasts, xanthoblasts and all chromatoblasts was normal through 24 hpf. Thus, the specification, migration and early differentiation of neural crest-derived chromatophores is unaffected by the *enz* mutation. Double mutant analysis of *enz* with *trpm7^b508^*, a mutation previously demonstrated to affect melanophores just prior to overt differentiation [67], revealed that embryos homozygous for mutations in both genes resembled *trpm7^b508^* single mutants with respect to the melanophore phenotype. This places *enz* genetically downstream of *trpm7^b508^* and, together with phenotypic characterization, suggests *enz* function is required for the terminal differentiation of chromatophores, the establishment of correct cell numbers and subsequently for the maintenance of chromatophore cell morphology.

### Potential functions of *enz* in zebrafish chromatophore development

The mechanism by which disruption of *enz* function affects chromatophore development is unclear. We have determined that it is unlikely that *enz* is required prior to overt differentiation. The fact that the numbers of differentiated chromatophores in *enz* mutants are reduced compared to wild-type embryos raises several possibilities. It is formally possible that incompletely differentiated chromatophores persist abnormally in *enz* mutants. That we have not detected comparatively more cells expressing chromatophore precursor markers and not pigment in *enz* mutants during embryogenesis argues against but does not rule out this possibility. On the other hand, embryonic chromatophores are believed to be mitotically active, although the proliferation rates of zebrafish chromatophores have not been determined. It is possible then, that *enz* mutations reduce chromatophore proliferation resulting in a smaller population of pigment cells. Conversely, *enz* mutations could result in an increase in chromatophore cell death that would result in reduced numbers of cells. Although TUNEL analysis at sequential stages of development between 16hpf and 30 hpf did not reveal detectable differences in melanophore cell death between mutant and wild type embryos (data not shown), there are several factors that prevent conclusions to be drawn from the experiments. First, TUNEL, as well as most other reliable methods to detect cell death in vivo are only useful in the instantaneous detection of apoptotic cells. Thus, if cell death were to occur at a low rate over an extended period, differences in cell death rate would be very difficult to detect. This would be especially true if, as is the case in *enz* mutants, the cell types of interest are only slightly reduced in number compared to control embryos. Nevertheless, it is potentially significant to note that multiple studies have shown that chromatophores differentiate and undergo apoptosis in response to factors that control chromatophore motile responses, that is, the redistribution of pigment organelles within chromatophores (see Sugimoto, 2002). Further, these factors are likely to utilize shared intracellular signaling pathways in different pigment cell types. Thus, the chromatophore phenotypic consequences and cell autonomous action of *enz* are at least consistent with a role for *enz* in such a pathway and the regulation of differentiation and apoptosis via such a pathway. Finally, in addition to the molecular identification of *enz* it will also be critical to determine whether any of the *enz* alleles we have identified result in loss of function. While the majority of chromatophores develop in *enz* mutants, the same situation is observed in most mutants and morphants affecting chromatophore development [4], [14], [15], [70], [71]. Further, the majority if not all chromatophores in more severe *enz* alleles exhibit altered morphology. Thus, it is possible that one or more of the *enz* alleles we have identified result in loss of function, but this will only be possible to ascertain by cloning the *enz* locus.

While the vast majority of homozygous *enz* larvae die after 2–3 weeks of development, a minority of individuals does survive to adulthood but were found to be significantly smaller than wild-type siblings. Because neural crest-derived chromatophores are not required for viability and are not thought to regulate growth [6], [7], [55], [72], *enz* must function in other cells required for growth and viability. Interestingly, there is abundant evidence demonstrating roles for pituitary hormone signaling systems in chromatophore development and growth control [42], [46], [73]. Notably, hormones known to regulate chromatophore morphology in teleosts specifically have been shown in mammals and fish to affect body weight and composition, as well [46]. Because our data indicate that *enz* acts cell autonomously, *enz* would be predicted to be a downstream effector if it acts in such a hormonal signaling pathway.

It is also potentially informative to note that recent reports have described mutants and morphants with live phenotypes similar to *enz* that disrupt genes involved in organellar biosynthesis and transport [70], [71], [74]. Although more in depth investigations into the nature of these pigmentation defects have not been described to date, such as whether organelle transport, chromatophore cell morpholgy or survival and proliferation are affected, morpholino-mediated knockdown of zebrafish *ATPase 6 subunit v0c* (*atp6v0c*) and *vacuolar protein sorting protein 18* (*vps18*) result in punctate melanophore appearance, apparent loss of xanthophores and reduced iridiphore numbers [71]. Similar phenotypes were observed in mutants for a variety of *ATP synthase* and *vacuolar protein sorting* genes generated in a viral insertion-based mutagenesis screen [70], [74]. We noted a predicted *vps35* ortholog assigned to chromosome 7 (http://www.sanger.ac.uk/), however, sequencing of the four *enz* alleles reported here did not reveal any molecular lesions in the *vps35* coding sequence. This does not rule out the possibility that *enz* encodes *vps35*, as lesions may be present in non-coding regulatory regions of this gene in *enz* mutants. Rawls and colleagues have identified a mutation linked to chromosome 7 and SSLP marker z10441 that phenotypically resembles *enz* [17]. However, *enz* mutants complement this mutation. Further, the genomic region in which the *enz* gene is located contains several other genes involved in organellar function that may be good candidates for the *enz* locus (http://zfin.org). Thus, it is also possible that the *enz* locus encodes a protein required for normal formation or activity of cellular organelles. Specifically in the case of *enz*, such a gene would have to regulate chromatophore cell morphology instead of or in addition to organelle synthesis and distribution. The characterization of *enz* mutant embryos and larvae presented here may provide clues to the mechanisms behind phenotypes described for organellar biosynthesis mutants and morphants described previously. In any case, the potential relevance of the speculation discussed here will be resolved with the molecular identification of the *enz* locus.

### Coordinated control of the late development of chromatophore sublineages

Although many loci have been identified that are required for crest-derived chromatophore development, relatively few of them affect all three chromatophore cell types found in zebrafish [14], [16], [17], [70]. Still fewer of these affect all chromatophore sublineages in the same way [14], [70], [74]. At the same time, other neural crest derivatives appear to develop normally in *enz* homozygotes. Together with our finding that the *enz* mutation acts cell autonomously with respect to melanophore development, this suggests that the *enz* locus does not encode a gene generally required by neural crest-derived cells, but rather a molecule intrinsic to chromatophores themselves. Our results strongly suggest that the differentiation and maintenance of cell morphology of all three zebrafish chromatophore cell types depends on *enz* and are thus at least partially coordinately regulated during terminal differentiation. However, since the development of all three chromatophore lineages is unaffected in *enz* mutants until late in their embryonic development and many mutants and identified genes selectively affect individual or subsets of chromatophore types [14], [16], [17], [70], [74], the regulation of earlier stages of chromatophore development is at least partially genetically distinct. Nevertheless, it will be interesting to determine when and to what degree the development of zebrafish chromatophore sublineages is regulated by common and distinct genes. The great numbers of zebrafish pigment mutants, including *enz*, will provide important contributions to ultimately determining the genetic regulation of chromatophore development and leading to potential insights into clinically relevant conditions in humans involving pigment cells. Further, many of these mutants are likely to provide valuable insights into the genetic regulation of color changes and light adaptation in fish and other organisms.

## Materials and Methods

### Zebrafish

Adult zebrafish and embryos were maintained in the Ohio State University zebrafish facility. Adults and embryos were reared at 28.5°C and embryos were staged based on morphological criteria, according to Kimmel et al., 1995. Mutant lines were maintained in the AB* and WIK backgrounds. Homozygous mutant embryos and wild-type siblings were obtained by crossing heterozygous carriers.

### Cell size quantification and analysis

Melanophores in 2 days post-fertilization (dpf) wild-type and *enz* embryos were imaged on a Zeiss Axioplan compound microscope. Individual cells were outlined and areas calculated using Zeiss Axioplan software with appropriate scalings. Iridiphores in 6 dpf larvae were imaged on a Leica dissecting microscope. Individual cells were outlined and areas calculated using Spot Advanced software with appropriate scalings. A minimum of 3 melanophores or iridiphores in at least 3 wild-type and 3 *enz* mutant individuals were utilized for each set of measurements. Standard errors of mean were calculated for wild-type and *enz* cell areas, and mean numbers were also subjected to a one-tailed t-test to determine whether differences observed between wild-type and *enz* chromatophore areas were statistically significant.

### Cell counts and statistics

Melanophores in 4 dpf larvae were counted in dorsal stripes. To better visualize distinct melanophores, wild-type and *enz* mutant larvae were placed in epinephrine (10 mg/ml) at 4 dpf for ∼10 minutes, that results in redistribution of melanosomes to the center of the cell body in wild-type melanophores (Johnson 1995, Rawls 2000). Larvae were fixed in 4% paraformaldehyde at 4°C overnight, rinsed and stored in 1∶1 PBS∶glycerol. Larvae were then deyolked, mounted on single bridge cover slips, and viewed on a Zeiss Axioplan microscope. Melanophores in the dorsal stripes were counted from somite 5 to somite 14. Iridiphores in 6 dpf larvae were counted in the dorsal and ventral stripes. Live fish were viewed under incident light using a Zeiss dissecting microscope. Iridiphores were counted posterior of the hindbrain in the dorsal stripe, and caudally from the posterior edge of the yolk ball in the ventral stripe. Standard errors of mean were calculated for wild-type and *enz* cell counts. The mean numbers of cells in wild-type and *enz* embryos were subjected to a one-tailed T-test to determine whether the decrease in cell numbers in *enz* mutant embryos compared to wild-type siblings was statistically significant.

### In situ hybridization

In situ hybridizations were performed as described by Thisse, et al. (1993) with minor modifications. A detailed protocol will be provided upon request. *mitfa* and *c-ret* cDNAs were provided by D. Raible [6], [75]. cDNA clones of *c-kit* and *fms* were obtained from D. Parichy [7], [28]. *dct* and *ednrb1* cDNA clones were provided by R. Kelsh [23], [37].

### Immunohistochemistry

Antibody labeling was performed as previously described [76]. 7 dpf larvae were cryo-sectioned onto gelatin-subbed slides and stored at −20°C overnight. All neurons were detected with monoclonal antibody 16A11 that recognizes neuron-specific Hu RNA binding proteins [15], [76], while DRG neurons and enteric neurons were subsequently identified by position within the larva. 16A11-immunoreactivity was detected using an Alexa red fluorescent secondary antibody (Molecular Probes). Sympathetic neurons were identified by detection of tyrosine hydroxylase (TH) using anti-TH polyclonal antibody (Pel-Freeze, Rogers, AZ). Neural crest-derived sympathetic neurons were subsequently assessed by location of TH^+^ cells within whole-mount wild-type and *endzone* larvae.

### Mosaic analysis

Genetic mosaics were produced using cell transplantation techniques [77]. Donor embryos obtained from AB* or heterozygous (*enz^+/b431^*) crosses were manually dechorionated and injected at the one to two cell stage with 2–5% lysinated rhodamine dextran (LRD, 10,000 MW, Molecular Probes) in 0.2 M KCl. To facilitate the identification of donor-derived melanophores, we utilized hosts homozygous for the *golden^b1^* mutation, which results in pale melanophores that are distinct from wild-type melanophores [7], [61]. Embryos were then allowed to develop to early blastula stages. 10–20 cells were transplanted from LRD-labeled donor embryos into unlabeled host embryos. Donor-host pairs were kept separate and allowed to develop to ≥32 hpf, then fixed in 4% paraformaldehyde at 4°C overnight. Donors and hosts were classified as either wild-type or mutant based on melanophore phenotype, and subsequently examined using a Zeiss Axioplan microscope with Nomarski optics, and a rhodamine filter to detect donor cells. Dark, punctate melanophores were scored as mutant, while dark, stellate melanophores were considered wildtype. Wild-type → wild-type and mutant → mutant transplants served as controls.

### Genetic mapping

*enz* alleles were maintained in the AB* background. For mapping purposes, *enz* carriers in this background were crossed to a wild-type WIK line, which is polymorphic to AB* [78]. *enz* carriers in the WIK background were then used to generate parthenogenetic diploid progeny by suppressing the second meiotic division with early pressure [66]. *enz* embryos and wild-type siblings were identified by live phenotype and used to obtain DNA. *enz* was initially placed on the zebrafish genomic map based on PCR amplification of simple sequence length polymorphisms (SSLPs) from diploid genomes [62]–[65]. *enz^b431^* was initially demonstrated to be linked to SSLP markers near the centromere of chromosome 7 based on the MGH mapping panel (http://zebrafish.mgh.harvard.edu/zebrafish/index.htm). *enz* was shown to cosegregate with z10441 (map position 36.7 cM), z11625 (map position 51.1 cM) and z6819 (map position 45.0 cM). Further linkage analysis was performed on *enz* using haploid genomes, or meioses [62]. This analysis confirms linkage to microsatellite marker z10441, and further defines a tentative interval for the *enz* locus distal to z6819 and z55887 and proximal to z8252 and z10441.
